# Association Between Aerobic Physical Activity and Hypertension Among U.S. Adults: A Cross-Sectional Analysis of NHANES (National Health and Nutrition Examination Survey) 2021-2023

**DOI:** 10.7759/cureus.116112

**Published:** 2026-09-11

**Authors:** Dylon J Wetzel, Alexandra "Xan" Nowakowski

**Affiliations:** 1 Medicine, Florida State University College of Medicine, Orlando, USA; 2 Geriatrics/Behavioral Sciences and Social Medicine, Florida State University College of Medicine, Orlando, USA

**Keywords:** aerobic physical activity, blood pressure, cardiovascular prevention, cross-sectional study, hypertension, lifestyle modification, nhanes, physical activity, population health, preventive medicine

## Abstract

Background

Hypertension continues to be a principal modifiable risk factor for cardiovascular disease and premature mortality in the United States. Current clinical guidelines recommend regular aerobic physical activity for the prevention and management of hypertension; however, contemporary population-level estimates from recent nationally representative data remain useful for evaluating whether established associations persist. This study evaluated the association between leisure-time aerobic physical activity and blood pressure-defined hypertension among U.S. adults.

Methods

We conducted a cross-sectional analysis of adults aged ≥18 years using data from the National Health and Nutrition Examination Survey (NHANES) 2021-2023. Participants with complete physical activity data and valid blood pressure measurements were included. Aerobic physical activity was categorized according to adherence to the Physical Activity Guidelines for Americans (≥150 minutes/week of moderate-intensity equivalent activity). Hypertension was defined as a mean systolic blood pressure ≥130 mmHg or a mean diastolic blood pressure ≥80 mmHg, based on up to three standardized oscillometric measurements. Antihypertensive medication use was not assessed and was not incorporated into this case definition. Survey-weighted multivariable logistic regression was performed to evaluate the association between meeting aerobic physical activity recommendations and hypertension after adjustment for age, sex, race/ethnicity, smoking status, alcohol consumption, household income, and educational attainment. Odds ratios (ORs) with 95% confidence intervals (CIs) were reported.

Results

The analytic sample included 6,123 participants, representing approximately 245 million U.S. adults. Overall, 3,254 participants (54.7% weighted) met recommended aerobic physical activity guidelines, and 2,465 participants (37.5% weighted) met criteria for hypertension. Hypertension prevalence was lower among adults meeting activity recommendations than among those who did not (1,210 participants, 34.4% weighted vs. 1,255 participants, 41.2% weighted). In unadjusted analyses, meeting aerobic activity guidelines was associated with lower odds of hypertension (OR, 0.75; 95% CI, 0.67-0.84). This association remained statistically significant after multivariable adjustment (adjusted OR, 0.79; 95% CI, 0.62-0.99; p=0.044). Age demonstrated the strongest association with hypertension, with adults aged ≥60 years having significantly greater odds of hypertension than those aged 18-39 years (adjusted OR, 2.35; 95% CI, 1.44-3.82). No significant effect modification was found across the subgroups examined.

Conclusions

In this nationally representative sample of U.S. adults, leisure-time aerobic physical activity meeting recommended levels was independently associated with lower adjusted odds of blood pressure-defined hypertension after adjustment for demographic, socioeconomic, and behavioral factors. Although the cross-sectional design precludes causal inference, physical activity was self-reported, and antihypertensive medication use was not incorporated into the hypertension case definition, these data support current guideline recommendations advocating regular aerobic physical activity as an important component of cardiovascular health.

## Introduction

Hypertension affects nearly half of U.S. adults and remains one of the leading modifiable risk factors for cardiovascular disease, stroke, chronic kidney disease, and premature mortality [1]. Despite the developments in pharmacologic therapy and evidence-based clinical guidelines, hypertension continues to impose a substantial public health burden due to its high prevalence, frequent underdiagnosis, and inadequate blood pressure control [1]. Because hypertension is both common and largely preventable, identifying effective lifestyle interventions continues to be a major priority for clinicians and public health professionals [2].

Regular aerobic physical activity is a mainstay of hypertension prevention and management [2]. Current guidelines from the American College of Cardiology, the American Heart Association, and the U.S. Department of Health and Human Services recommend that adults engage in at least 150 minutes of moderate-intensity or 75 minutes of vigorous-intensity aerobic physical activity each week to promote cardiovascular health and lessen the threat of chronic disease [2,3]. Aerobic exercise has been shown to lower blood pressure through multiple physiological mechanisms, including improved endothelial function, enhanced nitric oxide bioavailability, reduced sympathetic nervous system activity, improved arterial compliance, and favorable effects on body weight and insulin sensitivity [4,5]. Beyond its antihypertensive effects, routine physical exercise reduces overall cardiovascular morbidity and mortality and remains one of the most cost-effective interventions available for chronic disease prevention [6].

Although numerous studies have demonstrated an inverse relationship between physical activity and hypertension, including evidence from prospective cohort studies and longitudinal systematic reviews [4,7-9], the epidemiology of both physical activity and hypertension remains evolving. National estimates require periodic reassessment as population demographics, cardiovascular risk factors, and observance of physical activity recommendations change over time. Furthermore, recent nationally representative analyses utilizing the most current National Health and Nutrition Examination Survey (NHANES) data contribute to an evolving evidence base. Updated estimates are important for informing preventive counseling, supporting evidence-based clinical decision-making, and evaluating whether established associations persist in contemporary U.S. populations.

The National Health and Nutrition Examination Survey provides a unique opportunity to examine these relationships by utilizing standardized blood pressure measurements and nationally representative sampling methods [10]. By combining detailed health interviews with objective examination data, NHANES allows investigators to evaluate cardiovascular risk factors while factoring in the complex sampling design that yields estimates representative of the U.S. civilian, non-institutionalized population [10].

The objective of this study was to examine the association between leisure-time aerobic physical activity and blood pressure-defined hypertension among U.S. adults, using data from the NHANES 2021-2023. We hypothesized that adults meeting current aerobic physical activity recommendations would have lower odds of hypertension than those who did not meet guideline-recommended activity levels after adjustment for demographic, socioeconomic, and behavioral confounders.

This work was previously presented, in part, as an oral podium and poster presentation at the Florida Academy of Family Physicians Family Medicine Spring Forum (Orlando, FL, April 2026), and as a poster presentation at the American College of Physicians Florida Chapter Annual Residents & Medical Student Meeting (Orlando, FL, March 2026).

## Materials and methods

Study design

This cross-sectional study analyzed data from the 2021-2023 National Health and Nutrition Examination Survey (NHANES), a nationally representative survey conducted by the National Center for Health Statistics (NCHS) of the Centers for Disease Control and Prevention (CDC) [10]. NHANES uses a complex, multistage probability sampling design to collect health interview and examination data representative of the civilian, non-institutionalized U.S. population [10]. All participants provided written informed consent, and the NCHS Research Ethics Review Board approved the NHANES protocol [10]. Because this study comprised analysis of publicly available, de-identified data, it was considered exempt from additional institutional review board review.

Study population

Adults aged 18 years and older who participated in NHANES 2021-2023 were eligible for inclusion. Participants were included if complete information was available for self-reported aerobic physical activity and a valid blood pressure outcome. Of 11,933 total NHANES 2021-2023 participants of all ages, 6,337 adults aged ≥18 years with complete interview and mobile examination center (MEC) data were eligible. An additional 214 participants were excluded for missing physical activity or blood pressure data, yielding a final analytic sample of 6,123 participants, representing approximately 245 million U.S. adults after application of survey weights.

Exposure assessment

The primary exposure was leisure-time aerobic physical activity meeting current recommendations established by the U.S. Department of Health and Human Services. Physical activity information was obtained from the NHANES Physical Activity Questionnaire (source variables PAD790Q, PAD790U, PAD800, PAD810Q, PAD810U, PAD820; data file PAQ_L), which assesses the frequency and duration of moderate- and vigorous-intensity recreational aerobic activities performed during a typical week [10]. Total weekly aerobic activity was calculated according to guideline recommendations, with vigorous-intensity activity counted as twice the equivalent duration of moderate-intensity activity [3]. Participants were classified as meeting aerobic physical activity recommendations if they accumulated at least 150 minutes per week of moderate-intensity equivalent aerobic activity [3]. Participants who did not meet this threshold were classified as not meeting guideline recommendations. This exposure captures only leisure-time recreational activity and does not include occupational, household, or transportation-related physical activity, which may lead to an underestimation of total physical activity for some participants.

Outcome assessment

The primary outcome was hypertension. Blood pressure was measured by trained health professionals during the NHANES mobile examination center visit using uniform protocols (data file BPXO_L; source variables BPXOSY1-3, BPXODI1-3) [10]. Up to three oscillometric blood pressure measurements were obtained after an appropriate seated rest period, and the mean systolic and diastolic blood pressure values were calculated [10]. Hypertension was defined according to the 2017 American College of Cardiology/American Heart Association guideline as a mean systolic blood pressure of at least 130 mmHg or a mean diastolic blood pressure of at least 80 mmHg [2]. Antihypertensive medication use was not assessed in this analysis and was not incorporated into the case definition; consequently, this outcome represents measured blood pressure status independent of treatment and does not reflect the composite treated-and-untreated hypertension case definition used in some other studies.

Covariates

Possible confounding variables were selected a priori based on established associations with both physical activity and hypertension. Demographic variables included age (categorized as 18-39, 40-59, and ≥60 years), sex, and race/ethnicity. Race/ethnicity was collapsed from the more granular NHANES RIDRETH3 categories to Non-Hispanic White, Non-Hispanic Black, and Other, given limited sample sizes within individual granular categories (e.g., Mexican American, Other Hispanic, Non-Hispanic Asian) for stable subgroup estimation. Socioeconomic characteristics included educational attainment (college or higher vs. less than college; source variable DMDEDUC2) and household income, expressed as the poverty-income ratio (PIR; source variable INDFMPIR) and categorized as Low (PIR <1.3), Middle (PIR 1.3-3.5), or High (PIR >3.5). Behavioral variables included smoking status (current smoker vs. non-smoker; source variables SMQ020, SMQ040) and alcohol consumption. Alcohol consumption was categorized as Moderate (fewer than five drinks per drinking day, including non-drinkers) or Heavy (five or more drinks per drinking day), based on the NHANES ALQ130 variable, with Moderate as the reference category. Age, sex, race/ethnicity, education, and income were drawn from the NHANES Demographics file (DEMO_L); smoking status from the Smoking-Cigarette Use file (SMQ_L); and alcohol consumption from the Alcohol Use file (ALQ_L). These variables were included in multivariable analyses to reduce confounding and better estimate the independent association between aerobic physical activity and hypertension.

Statistical analysis

All analyses incorporated NHANES examination sample weights, strata, and primary sampling units to adjust for the complex multistage sampling design and generate nationally representative estimates [10], consistent with CDC/NCHS guidance for the NHANES August 2021-August 2023 combined cycle [11]. Descriptive statistics were used to summarize participant characteristics and to estimate the prevalence of hypertension by aerobic physical activity status. Bivariate associations between categorical variables, including aerobic physical activity status and hypertension and between aerobic physical activity status and selected covariates, were assessed using survey-weighted Rao-Scott χ² tests (design-adjusted F-statistics).

Survey-weighted univariable logistic regression was used to evaluate the unadjusted association between meeting aerobic physical activity recommendations and hypertension, and a survey-weighted multivariable logistic regression model was subsequently fitted to adjust for age category, sex, race/ethnicity, smoking status, alcohol consumption, household income, and educational attainment. A sequential (block-wise) model-building approach was also used to characterize the contribution of covariate groups to attenuation of the exposure estimate. Effect modification was assessed by including multiplicative interaction terms between aerobic physical activity and each covariate individually in the fully adjusted survey-weighted logistic regression model, with statistical significance evaluated using survey-adjusted Wald tests; stratum-specific adjusted odds ratios were also estimated for subgroups defined by sex, age category, and income level. Results are reported as odds ratios (ORs) with corresponding 95% confidence intervals (CIs).

The Lumono.ai (Lumono Labs, Inc., Richardson, Texas, United States) Research Platform was used for data extraction, data cleaning, variable transformation, and statistical analysis. Lumono.ai AI-assisted functionality was used to generate R code and facilitate the statistical analyses. The survey-weighted analyses were performed using R version 4.5.0 (R Foundation for Statistical Computing, Vienna, Austria) and the survey package (version 4.4-8) to take into account the complex NHANES survey design, including sampling weights, strata, and primary sampling units. The authors selected statistical methods, and interpretation of the results was performed by the authors. The generated analysis code was not independently preserved for full replication. As a confirmation step, the study population, exposure, and outcome variable definitions were independently reconstructed within the Lumono.ai platform; this reconstruction exactly reproduced the reported analytic sample size (n=6,123) and outcome distribution (2,465 hypertensive, 3,658 non-hypertensive participants), supporting the accuracy of the underlying cohort construction. A formal multicollinearity diagnostic (e.g., variance inflation factors) for the fully adjusted model was not performed and is noted as a limitation.

## Results

A total of 6,123 adults met the inclusion criteria, representing an estimated 245.2 million non-institutionalized U.S. adults after application of NHANES survey weights. Overall, 54.7% of participants met the recommended aerobic physical activity guidelines (≥150 minutes/week of moderate-intensity equivalent activity), and 37.5% met the study definition of hypertension (Table 1).

**Table 1:** Participant Characteristics by Physical Activity Level Values are presented as unweighted participant counts (n) and survey-weighted percentages (%). The total analytical cohort included 6,123 participants. Activity levels were defined as <150 minutes/week versus ≥150 minutes/week of moderate-intensity equivalent aerobic activity. All percentages take into account the complex NHANES survey design. NHANES: National Health and Nutrition Examination Survey.

Characteristic	Does not meet activity (<150 min/week)	Meets activity (≥150 min/week)
Age Category		
18-39 years	680 (30.2%)	1,070 (42.8%)
40-59 years	827 (35.7%)	855 (30.0%)
60+ years	1,362 (34.1%)	1,329 (27.1%)
Sex		
Female	1,735 (57.1%)	1,612 (46.2%)
Male	1,134 (42.9%)	1,642 (53.8%)
Race/Ethnicity		
Non-Hispanic White	1,653 (62.5%)	1,930 (65.5%)
Non-Hispanic Black	361 (11.9%)	382 (11.2%)
Other	855 (25.6%)	942 (23.3%)
Smoking Status		
Non-smoker	775 (60.6%)	777 (62.7%)
Current smoker	476 (39.4%)	408 (37.3%)
Income		
Low (<1.3)	628 (23.6%)	574 (17.9%)
Middle (1.3-3.5)	997 (39.2%)	1,016 (35.8%)
High (>3.5)	868 (37.2%)	1,248 (46.2%)
Education		
Less than college	1,116 (71.7%)	872 (60.6%)
College or higher	1,674 (28.3%)	2,198 (39.4%)
Hypertension Status		
No hypertension	1,614 (58.8%)	2,044 (65.6%)
Has hypertension	1,255 (41.2%)	1,210 (34.4%)

Compared with participants who did not meet aerobic activity recommendations, those meeting guideline-recommended activity levels were younger, more likely to be male, had higher household income, and were more likely to have attained a college education. Specifically, 42.8% of physically active adults were aged 18-39 years compared with 30.2% of inactive adults, while 53.8% of active participants were male compared with 42.9% of inactive participants. College educational attainment was more common among active participants (39.4% vs. 28.3%), and a greater proportion belonged to the highest income category (46.2% vs. 37.2%). The prevalence of hypertension was lower among adults meeting aerobic physical activity recommendations than among those who did not (34.4% vs. 41.2%).

A survey-weighted Rao-Scott χ² test demonstrated a significant association between physical activity and hypertension (F = 36.14, df = 1, 15; p < 0.001; Table 2). Age, sex, race/ethnicity, household income, and educational attainment were also significantly associated with physical activity, supporting their inclusion as possible confounders in multivariable analyses, whereas smoking status and alcohol consumption were not.

**Table 2:** Survey-Weighted Rao-Scott χ² Analysis of the Association Between Aerobic Physical Activity and Hypertension Values are presented as unweighted participant counts (n) and survey-weighted percentages (%). Percentages are weighted to take into account the complex NHANES survey design, including examination sample weights, strata, and primary sampling units. The Rao-Scott χ² test yielded an F statistic of 36.14 (df = 1, 15), p < 0.001. NHANES: National Health and Nutrition Examination Survey.

Aerobic physical activity status	No hypertension, n (%)	Hypertension, n (%)	Total, n
Does not meet guidelines (<150 min/week)	1,614 (58.8%)	1,255 (41.2%)	2,869
Meets guidelines (≥150 min/week)	2,044 (65.6%)	1,210 (34.4%)	3,254
Total	3,658	2,465	6,123

In the unadjusted survey-weighted logistic regression model, meeting aerobic physical activity recommendations was associated with significantly lower odds of hypertension (OR, 0.75; 95% CI, 0.67-0.84; p < 0.001; Table 3). After adjustment for age category, sex, race/ethnicity, smoking status, alcohol consumption, household income, and educational attainment, meeting aerobic activity levels remained independently associated with lower odds of hypertension (adjusted OR, 0.79; 95% CI, 0.62-0.99; p = 0.044), corresponding to 21% lower adjusted odds of hypertension. This estimate's confidence interval approaches the null value, denoting a relatively imprecise association that should be interpreted with appropriate caution.

**Table 3:** Unadjusted Survey-Weighted Logistic Regression Analysis of the Association Between Aerobic Physical Activity and Hypertension The analytical sample included 6,123 adults. The reference category was participants who did not meet recommended aerobic activity guidelines (<150 minutes/week). The odds ratio represents the odds of hypertension among those participants who meet aerobic activity guidelines compared with those who do not. Estimates were obtained using survey-weighted logistic regression incorporating NHANES examination sample weights, strata, and primary sampling units. NHANES: National Health and Nutrition Examination Survey.

Variable	OR	95% CI	p-value
Meets aerobic activity guidelines (≥150 min/week)	0.75	0.67-0.84	<0.001

Among covariates included in the fully adjusted model, age demonstrated the strongest association with hypertension. Compared with adults aged 18-39 years, those aged 40-59 years had nearly twice the odds of hypertension (OR, 1.93; 95% CI, 1.31-2.84; p = 0.009), while adults aged 60 years and older had more than twice the odds (OR, 2.35; 95% CI, 1.44-3.82; p = 0.008). None of the remaining demographic, socioeconomic, or behavioral covariates were statistically significant in the fully adjusted model (Table 4).

**Table 4:** Survey-Weighted Logistic Regression Analysis of Hypertension Odds ratios (ORs) and 95% confidence intervals (CIs) were estimated using survey-weighted multivariable logistic regression incorporating NHANES sample weights, strata, and primary sampling units. The analysis included 6,123 adults. The reference category for aerobic physical activity was not meeting recommended activity guidelines (<150 minutes/week). The model was adjusted for age category, sex, race/ethnicity, smoking status, alcohol consumption, household income, and educational attainment. NHANES: National Health and Nutrition Examination Survey.

Variable	Adjusted OR	95% CI	p-value
Meets aerobic activity guidelines (≥150 min/week)	0.79	0.62-0.99	0.044
Age 40-59 y vs 18-39 y	1.93	1.31-2.84	0.009
Age ≥60 y vs 18-39 y	2.35	1.44-3.82	0.008
Male sex vs female	1.03	0.66-1.60	0.869
Non-Hispanic Black vs White	1.77	0.64-4.89	0.194
Other race vs White	0.69	0.35-1.35	0.199
Current smoker vs non-smoker	0.78	0.51-1.19	0.178
Heavy alcohol use vs moderate	2.44	0.64-9.40	0.139
Middle income vs low	0.95	0.45-2.01	0.863
High income vs low	0.89	0.45-1.74	0.651
College education vs less than college	0.91	0.62-1.34	0.549

A block-wise sequential modeling approach showed that adjustment for demographic factors (age, sex, race/ethnicity, income, education) attenuated the exposure odds ratio from 0.75 (unadjusted) to 0.86; subsequent adjustment for smoking status yielded an odds ratio of 0.89; and the fully adjusted model, additionally incorporating alcohol consumption, yielded the reported estimate of 0.79. This pattern indicates that demographic factors accounted for the majority of observed attenuation, consistent with age and sex representing the primary confounders identified in Table 4.

No statistically significant effect modification was observed by sex, age category, race/ethnicity, educational attainment, or household income (all interaction p > 0.10), indicating that the association between aerobic physical activity and hypertension was generally consistent across the subgroups examined. Stratified analyses, presented in Table 5, showed a statistically significant protective association among high-income participants specifically (OR, 0.65; 95% CI, 0.44-0.98; p = 0.042), which was not observed in the low- or middle-income strata. The association was directionally stronger, though not statistically significant, among male participants (OR, 0.64; 95% CI, 0.39-1.06; p = 0.070) compared with female participants (OR, 1.02; 95% CI, 0.67-1.55; p = 0.904), and among younger adults aged 18-39 years (OR, 0.54; 95% CI, 0.24-1.21; p = 0.111) compared with older age groups.

**Table 5:** Stratum-Specific Adjusted Odds Ratios for the Association Between Aerobic Physical Activity and Hypertension Each stratum-specific model was adjusted for the remaining covariates in the fully adjusted model (age, sex, race/ethnicity, smoking status, alcohol consumption, income, and education, excluding the stratifying variable). PIR: poverty-income ratio.

Stratum	N	Adjusted OR	95% CI	p-value
Female	3,347	1.02	0.67-1.55	0.904
Male	2,776	0.64	0.39-1.06	0.07
Age 18-39 years	1,750	0.54	0.24-1.21	0.111
Age 40-59 years	1,682	0.69	0.37-1.29	0.199
Age 60+ years	2,691	1.16	0.65-2.09	0.547
Income: Low (PIR < 1.3)	1,225	0.78	0.45-1.34	0.302
Income: Middle (PIR 1.3-3.5)	1,990	1.02	0.58-1.79	0.937
Income: High (PIR > 3.5)	2,116	0.65	0.44-0.98	0.042

## Discussion

This nationally representative cross-sectional analysis of NHANES 2021-2023 data found that U.S. adults engaging in leisure-time aerobic physical activity at recommended levels had significantly lower odds of blood pressure-defined hypertension than those who did not. After adjustment for demographic, socioeconomic, and behavioral factors, meeting the guideline-recommended level of aerobic activity was independently associated with 21% lower adjusted odds of hypertension; the confidence interval (0.62-0.99) approaches the null, denoting a relatively imprecise association that warrants reserved interpretation and replication in future work. These results are broadly consistent with public health recommendations endorsing regular aerobic physical activity as a component of cardiovascular health [2,3].

Our findings are consistent with a large body of literature demonstrating an inverse relationship between physical activity and blood pressure [4,7-9]. Previous epidemiologic studies and meta-analyses have consistently reported that routine aerobic exercise is associated with lower blood pressure and reduced risk of hypertension [7-9]. Clinical trials have similarly shown that aerobic exercise lowers both systolic and diastolic blood pressure among individuals with and without established hypertension [4,7]. The present study expands this evidence by demonstrating that this association persists in a contemporary sample of U.S. adults using NHANES 2021-2023 data, reflecting contemporary physical activity patterns and hypertension prevalence.

Multiple biological mechanisms may explain the observed association between aerobic physical activity and reduced odds of hypertension. Routine aerobic exercise improves endothelial function by increasing nitric oxide bioavailability, consequently enhancing vasodilation and reducing peripheral vascular resistance [4,5]. Exercise also decreases sympathetic nervous system activity while increasing parasympathetic tone, therefore reducing resting blood pressure [4]. In addition, habitual physical activity improves arterial compliance, reduces systemic inflammation and oxidative stress, promotes favorable body composition, and enhances insulin sensitivity [4,5]. Collectively, these physiologic adaptations improve vascular function and long-term blood pressure regulation, providing biological plausibility for the association observed in this study.

Age remained the strongest independent predictor of hypertension in the adjusted model, with adults aged 60 years and older demonstrating more than twice the odds of hypertension compared with adults aged 18-39 years. This finding is consistent with the well-established increase in hypertension prevalence with advancing age [1] and likely reflects age-related vascular remodeling, increased arterial stiffness, and cumulative exposure to cardiovascular risk factors [12]. Although other demographic and socioeconomic variables were included in the multivariable model, none remained statistically significant after adjustment, suggesting that age accounted for much of the observed variation in hypertension risk within this nationally representative sample.

No statistically significant interaction was observed between aerobic physical activity and sex, age, race/ethnicity, educational attainment, or household income, indicating that the inverse association between physical activity and hypertension was generally consistent across the examined subgroups. Stratified analyses showed that the protective association reached statistical significance specifically among high-income participants (OR, 0.65; 95% CI, 0.44-0.98; p = 0.042), but not among low- or middle-income participants, and was directionally, though not statistically significantly, stronger among male and younger participants. Because the corresponding interaction terms did not reach statistical significance, such subgroup differences should be interpreted as hypothesis-generating rather than confirmatory.

From a clinical perspective, these results uphold the value of lifestyle counseling during routine primary care encounters. Aerobic physical activity represents a low-cost, accessible, and evidence-based intervention [2,3] that offers benefits extending beyond blood pressure reduction, including improved cardiovascular fitness, weight management, glycemic control, and overall cardiovascular health [6]. Given the substantial burden of hypertension in the United States [1], increasing adherence to recommended physical activity guidelines may be an effective population-level strategy [3] to reduce cardiovascular disease risk and promote beneficial long-term health outcomes.

Several limitations should be considered when interpreting these results. First, the cross-sectional design precludes determination of temporal relationships or causal inference. Second, aerobic physical activity was assessed through self-reported questionnaire data limited to leisure-time recreational activity and is therefore subject to recall bias, misclassification, and underestimation of total physical activity relative to measures that also capture occupational, household, or transportation-related activity. Third, the hypertension case definition in this analysis was based solely on measured blood pressure; antihypertensive medication use was not assessed or incorporated into the case definition. This differs from the composite treated-and-untreated hypertension case definition used in some other studies and may result in the misclassification of treated, blood pressure-controlled individuals as non-hypertensive; this could affect both the prevalence estimate and the exposure-outcome association if medication use correlates with physical activity level and should be considered when interpreting the reported estimates. Fourth, although multiple demographic, socioeconomic, and behavioral factors were included in the adjusted analyses, residual confounding from unmeasured variables, including dietary intake, medication adherence, family history, and comorbid conditions, cannot be excluded; a formal multicollinearity diagnostic for the fully adjusted model was not performed. Fifth, of 6,337 eligible adults, 214 were excluded for missing exposure or outcome data; the exact apportionment of this exclusion between missing physical activity and missing blood pressure data could not be determined with certainty from the retained analytic dataset. Finally, because NHANES represents the civilian, non-institutionalized U.S. population, these results may not be generalizable to institutionalized populations or individuals residing outside the United States.

In spite of these limitations, this study has several important strengths. NHANES uses rigorous sampling methods [10] to produce nationally representative estimates of the U.S. adult population, consequently strengthening the generalizability of the findings. Additionally, blood pressure measurements were obtained by applying standardized examination protocols [10] rather than self-report, hence reducing outcome misclassification. Furthermore, the use of survey-weighted multivariable logistic regression accounted for the complex sampling design. It adjusted for multiple potential confounders, consequently strengthening the validity of the observed association, and an independent reconstruction of the study cohort reproduced the reported analytic sample size and outcome distribution, supporting the accuracy of the underlying data extraction and cohort construction.

## Conclusions

In this nationally representative cross-sectional analysis of NHANES 2021-2023 data, adults engaging in leisure-time aerobic physical activity at recommended levels had significantly lower adjusted odds of blood pressure-defined hypertension than those who did not, even after adjustment for demographic, socioeconomic, and behavioral factors. Although the cross-sectional design precludes causal inference, physical activity was self-reported, and antihypertensive medication use was not incorporated into the hypertension case definition, these results are consistent with the well-established association between regular aerobic physical activity and cardiovascular health. Promoting adherence to current physical activity recommendations may represent an effective, accessible strategy for reducing the burden of hypertension and improving population cardiovascular health. Future prospective and interventional studies, incorporating medication use into the hypertension case definition, are needed to further investigate the relationship between aerobic physical activity and hypertension and to identify interventions that improve long-term adherence to recommended activity levels.
